# Characterization of demethylating DNA glycosylase ROS1 from Nicotiana tabacum L.

**DOI:** 10.18699/VJGB-22-41

**Published:** 2022-07

**Authors:** D.V. Petrova, N.V. Permyakova, I.R. Grin, D.O. Zharkov

**Affiliations:** Institute of Chemical Biology and Fundamental Medicine of the Siberian Branch of the Russian Academy of Sciences, Novosibirsk, Russia; Institute of Cytology and Genetics of the Siberian Branch of the Russian Academy of Sciences, Novosibirsk, Russia; Institute of Chemical Biology and Fundamental Medicine of the Siberian Branch of the Russian Academy of Sciences, Novosibirsk, Russia; Institute of Chemical Biology and Fundamental Medicine of the Siberian Branch of the Russian Academy of Sciences, Novosibirsk, Russia Novosibirsk State University, Novosibirsk, Russia

**Keywords:** epigenetic demethylation, 5-methylcytosine, 5-hydroxymethylcytosine, DNA glycosylases, REPRESSOR OF SILENCING 1, Nicotiana tabacum, эпигенетическое деметилирование, 5-метилцитозин, 5-гидроксиметилцитозин, ДНК-гликозилазы, REPRESSOR OF SILENCING 1, Nicotiana tabacum

## Abstract

One of the main mechanisms of epigenetic regulation in higher eukaryotes is based on the methylation of cytosine at the C5 position with the formation of 5-methylcytosine (mC), which is further recognized by regulatory proteins. In mammals, methylation mainly occurs in CG dinucleotides, while in plants it targets CG, CHG, and CHH sequences (H is any base but G). Correct maintenance of the DNA methylation status is based on the balance of methylation, passive demethylation, and active demethylation. While in mammals active demethylation is based on targeted regulated damage to mC in DNA followed by the action of repair enzymes, demethylation in plants is performed by specialized DNA glycosylases that hydrolyze the N-glycosidic bond of mC nucleotides. The genome of the model plant Arabidopsis thaliana encodes four paralogous proteins, two of which, DEMETER (DME) and REPRESSOR OF SILENCING 1 (ROS1), possess 5-methylcytosine-DNA glycosylase activity and are necessary for the regulation of development, response to infections and abiotic stress and silencing of transgenes and mobile elements. Homologues of DME and ROS1 are present in all plant groups; however, outside A. thaliana, they are poorly studied. Here we report the properties of a recombinant fragment of the ROS1 protein from Nicotiana tabacum (NtROS1), which contains all main structural domains required for catalytic activity. Using homologous modeling, we have constructed a structural model of NtROS1, which revealed folding characteristic of DNA glycosylases of the helix– hairpin–helix structural superfamily. The recombinant NtROS1 protein was able to remove mC bases from DNA, and the enzyme activity was barely affected by the methylation status of CG dinucleotides in the opposite strand. The enzyme removed 5-hydroxymethylcytosine (hmC) from DNA with a lower eff iciency, showing minimal activity in the presence of mC in the opposite strand. Expression of the NtROS1 gene in cultured human cells resulted in a global decrease in the level of genomic DNA methylation. In general, it can be said that the NtROS1 protein and other homologues of DME and ROS1 represent a promising scaffold for engineering enzymes to analyze the status of epigenetic methylation and to control gene activity.

## Abstract (ru)

Один из главных механизмов эпигенетической регуляции у высших эукариот основан на метилировании цитозина по положению C5 с образованием 5-метилцитозина (mC), который далее узнается регуляторными белками. У млекопитающих метилирование преимущественно протекает в динуклеотидах CG, тогда как у растений его мишенью служат последовательности CG, CHG и CHH (H – любое основание, кроме G). Корректное поддержание статуса метилирования ДНК требует баланса процессов метилирования, пассивного и активного деметилирования. В то время как у млекопитающих активное деметилирование происходит за счет направленного регулируемого повреждения mC в ДНК с последующим действием ферментов репарации, у растений функции деметилирования выполняют специализированные ДНК-гликозилазы, гидролизующие N-гликозидную связь mC-нуклеотидов. Геном модельного растения Arabidopsis thaliana кодирует четыре паралогичных белка, два из которых – DEMETER (DME) и REPRESSOR OF SILENCING 1 (ROS1) – обладают 5-метилцитозин-ДНК-гликозилазной активностью и необходимы для регуляции развития, ответа на инфекции и абиотический стресс и сайленсинга трансгенов и мобильных элементов. Гомологи DME и ROS1 присутствуют во всех группах растений, однако за пределами A. thaliana исследованы крайне слабо. В статье приведены результаты изучения свойств рекомбинантного фрагмента белка ROS1 из Nicotiana tabacum (NtROS1), содержащего основные структурные домены, необходимые для каталитической активности. Методами гомологичного моделирования была построена структурная модель NtROS1, в которой выявлена укладка, характерная для ДНК-гликозилаз структурного суперсемейства «спираль–шпилька–спираль». Рекомбинантный белок NtROS1 был способен удалять из ДНК основания mC, причем активность фермента слабо зависела от статуса метилирования CG-динуклеотидов в противоположной цепи. С меньшей эффективностью фермент удалял из ДНК 5-гидроксиметилцитозин (hmC), проявляя минимальную активность при наличии mC в противоположной цепи. При экспрессии гена NtROS1 в клетках человека в культуре происходило глобальное снижение уровня метилирования геномной ДНК. В целом можно сказать, что белок NtROS1 и другие гомологи DME и ROS1 представляют собой многообещающую основу для инженерии ферментов с целью анализа статуса эпигенетического метилирования и управления активностью генов.

## Introduction

DNA methylation is a dedicated mechanism of gene regulation, especially developed in higher eukaryotes. The 5-methylcytosine (mC) nucleobase, formed by cytosine methylation at the C5 position, serves as a reversible epigenetic mark that plays an important role in the control of gene expression and the protection of the genome from mobile elements. DNA methylation occurs in a wide range of multicellular eukaryotes; however, its significance and functions in these organisms differ greatly (Lee et al., 2010; Zemach, Zilberman, 2010). For example, in mammals, methylation most often occurs in CpG dinucleotides, while in plants, a significant proportion of mC occurs in trinucleotides CHG and CHH (where H is “not G”). The consequences of mC for gene activity are mainly mediated by proteins containing methyl-binding domains that form complexes with histone deacetylases or themselves have the activity of histone-specific methyltransferases, chromatin remodeling factors, etc., which leads to chromatin condensation and transcription suppression (Ballestar, Wolffe, 2001; Baubec et al., 2013). Recent studies have shown that an oxidized derivative of 5-methylcytosine, 5-hydroxymethylcytosine (hmC), also plays an epigenetic role in the mammalian genome (Branco et al., 2011). Unlike mC, hmC is enriched in promoters and bodies of actively expressed genes and is considered an activating epigenetic marker (Pastor et al., 2011; Yu et al., 2012)

Correct methylation of various sites in the genome is extremely important, since the transcriptional activity of genes depends on it. Errors in DNA methylation can have grave consequences. In particular, in humans, global DNA demethylation or hypermethylation of tumor suppressor genes serve as cancer markers. Maintaining the status of DNA methylation in cells requires a balance of methylation and active and passive demethylation. The mechanisms of active demethylation of the genomic DNA in higher eukaryotes have only been discovered in the last decade. In mammals, active demethylation is initiated by regulated damage to mC, which can occur in two ways: either deamination of mC to T by AID/APOBEC enzymes, or oxidation of mC to hmC and further derivatives (5-formylcytosine and 5-carboxycytosine) by TET family dioxygenases (Pastor et al., 2013; Bochtler et al., 2017; Wu, Zhang, 2017). The modified bases are further perceived by cellular repair systems as damaged and are removed by the DNA base excision repair pathway.

Unlike mammals, plants have unique enzymes that directly hydrolyze N-glycosidic bonds of mC nucleotides. These DNA glycosylases – DEMETER (DME) and REPRESSOR OF SILENCING 1 (ROS1, also known as DEMETER-LIKE 1 or DML1) (Choi Y. et al., 2002; Gong et al., 2002; Agius et al., 2006; Morales-Ruiz et al., 2006) – are involved in the regulation of the methylation status of the sites in the plant genome that determine gene imprinting during paternal or maternal inheritance and silence or activate specific promoters during plant development and stress response (Li Y. et al., 2018; Parrilla-Doblas et al., 2019; Roldán-Arjona et al., 2019). After removal of mC, the resulting apurinic-apyrimidinic site (AP site) is cleaved either by the enzyme’s own AP lyase activity or by the AP endonucleases APE1L or ARP, then a normal nucleotide is incorporated by one of the DNA polymerases, and the nick is ligated by the LIG1 DNA ligase. Interestingly, DME and ROS1 can also excise hmC, which is not regarded as an epigenetic base in plants (Jang et al., 2014). The localization of demethylation by DME/ROS1 is regulated by small RNAs that bind to the enzyme itself or to the protein complex that contains it (Penterman et al., 2007; Li X. et al., 2012).

In addition to DME and ROS1, the genome of the model plant Arabidopsis thaliana contains three more genes that are homologous to DME and ROS1: DEMETER-LIKE 2 (DML2), DEMETER-LIKE 3 (DML3), and AT3G47830, which has not been characterized thus far except for participation of DML2 and DML3 in maintaining correct DNA methylation (Ortega- Galisteo et al., 2008; Le et al., 2014). All these proteins belong to the DNA helix–hairpin–helix (HhH) structural superfamily of DNA glycosylases. Other members of this superfamily are involved in the removal of oxidized, alkylated and deaminated nucleobases from the genome (Zharkov, 2008; Fedorova et al., 2010). DME/ROS1 enzymes attract attention as potential tools for targeted regulation of gene activity: for example, the possibility of targeted DNA demethylation in human cells by A. thaliana ROS1 (AtROS1) fused to the RNA-guided Cas9 protein has been shown (Devesa-Guerra et al., 2020), and A. thaliana DME (AtDME) was used to analyze the level of mC in genomic DNA (Choi W.L. et al., 2021).

In plants other than A. thaliana, there were few studies on the role of DME-like proteins in active epigenetic demethylation; some data exist for rice, wheat, barley, and tomato (Ono et al., 2012; Wen et al., 2012; Kapazoglou et al., 2013; Liu et al., 2015). In 2007, a ROS1 homolog from Nicotiana tabacum (NtROS1) was cloned, and the recombinant protein produced in insect cell culture was shown to cleave methylated tobacco genomic DNA (Choi C.-S., Sano, 2007). None of these studies included a detailed biochemical characterization of the protein. We have previously shown that the NtROS1 fragment corresponding to the minimal catalytically active AtROS1 fragment has the activity of 5-methylcytosine-DNA glycosylase (Gruber et al., 2018).

Here, in view of the potential value of plant demethylation enzymes as tools for genetic technologies, we characterize the substrate specificity of the recombinant NtROS1 catalytic fragment on mC and hmC in different contexts of methylated CpG dinucleotides and show that the expression of NtROS1 in human cells causes a global decrease in DNA methylation.

## Materials and methods

ProtoScript II reverse transcriptase, Q5 Hot Start High-Fidelity DNA polymerase, Escherichia coli uracil DNA glycosylase, ClaI and SacI restriction endonucleases were purchased from New England Biolabs (USA), and bacteriophage T4 polynucleotide kinase, from Biosan (Novosibirsk, Russia). Oligonucleotides listed in the Table were synthesized at the SB RAS ICBFM Laboratory of Biomedical Chemistry using commercially available prosphoramidites (Glen Research, USA). If necessary, the oligonucleotides were 32P-labeled at the 5′-end using γ[32P]ATP (SB RAS ICBFM Laboratory of Biotechnology) and T4 polynucleotide kinase.

**Tab. 1.**
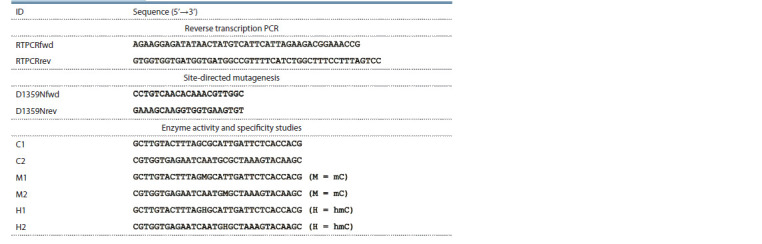
Oligonucleotides used in this work

To build a model of the NtROS1 catalytic domain in the Swiss-Model program (Waterhouse et al., 2018), the AtDME (AF-Q8LK56-F1-model_v1) and AtROS1 (AF-Q9SJQ6-F1- model_v1) templates from the AlphaFold database (Jumper et al., 2021) were used.

To obtain a catalytically inactive NtROS1 with the Asp1359Asn substitution, a pLATE31 plasmid with an insert encoding the catalytically active NtROS1 fragment (amino acid residues 754–1796) (Gruber et al., 2018) was mutagenized using primers D1359Nfwd and D1359Nrev (see the Table) and the Q5 Site-Directed Mutagenesis Kit (New England Biolabs). The mutation was confirmed by Sanger sequencing. Wild-type NtROS1 and NtROS1 D1359N were overproduced and purified as described previously (Gruber et al., 2018).

To study the activity of NtROS1, double-stranded substrates were obtained by annealing oligonucleotides C1, C2, M1, M2, H1, and H2 (see the Table). The reaction mixture contained 50 nM substrate, 50 mM Tris–HCl (pH 8.0), 1 mM EDTA, 1 mM DTT, 0.1 % bovine serum albumin, and 100 nM NtROS1. The mixture was incubated at 37 °C; aliquots were withdrawn at various times (2–300 min) and mixed with an equal volume of the stop solution (80 % formamide, 20 mM EDTA, 0.1 % xylene cyanol, 0.1 % bromophenol blue). If necessary, the aliquots were preheated for 2 min at 95 °C in the presence of 0.1 M NaOH and neutralized with an equimolar amount of HCl. The reaction products were resolved by electrophoresis in a 20 % polyacrylamide gel containing 7.2 M urea, visualized by prosphorimaging using the Typhoon FLA 9500 system (GE Healthcare, USA), and quantified using the Quantity One v4.6.3 software (Bio-Rad Laboratories, USA). The apparent rate constants were determined using the SigmaPlot v11.0 software (Systat Software, USA) by nonlinear regression to the equation [P] = [P]max(1 – e−kt ), where [P] is product concentration, [P]max is the maximum product concentration, k is the reaction rate constant, and t is time.

To assess the status of global DNA methylation upon NtROS1 expression in human cells, wild-type and D1359N NtROS1 coding sequences were cloned into the pIRES-eGFPpuro plasmid (Clontech, USA) at the SacI and ClaI restriction sites. HEK293 Phoenix cells (1.2·106) were transfected with 5 μg of the plasmid by the calcium phosphate method and grown in a monolayer in DMEM with 10 % fetal calf serum (HyClone, USA). After 24 and 48 h, the medium was changed with the addition of 3 μg/ml puromycin. Transfection efficiency was determined by flow cytometry (NovoCyte 3000, ACEA Biosciences, USA) by detection of the fluorescence of eGFP encoded by the same plasmid. NtROS1 expression in the transfected cells was confirmed by reverse transcription PCR using β-actin mRNA as a control. Genomic DNA was isolated from the cells (5·106) using a QIAamp DNA Mini Kit (Qiagen, the Netherlands), and the relative content of mC was determined using anti-mC antibodies (MethylFlash Methylated DNA Quantification Kit, EpiGentek, USA). The results were compared using Student’s t-test.

## Results and discussion

**Fig. 1.**
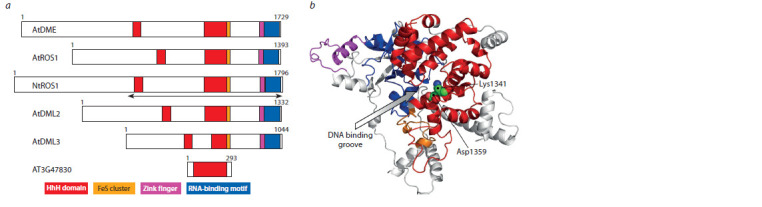
Structure of NtROS1. a, Organization of DME/ROS1 family proteins. Arrows mark the catalytic fragment of NtROS1. b, Model of the NtROS1 catalytic fragment (long disordered regions are not shown). The HhH domain is shown in red in both figures, the FeS cluster is in orange, the CXXC type zinc finger is in purple, and the RRM RNA-binding motif is in blue.

Plant mC-specific DNA glycosylases are proteins of considerable size: for example, AtDME and AtROS1, as well as their DML2 and DML3 paralogs, are over 1000 amino acid residues long (Fig. 1). The extended N-terminal regions of these polypeptides are unstructured, although some of their parts are necessary for enzyme activity. The C-terminal regions contain a conserved HhH catalytic domain and an iron-sulfur cluster (FeS cluster), characteristic of many DNA glycosylases that recognize oxidative DNA damage, as well as an RNAbinding motif (RNA Recognition Motif, RRM) and a CXXC type permuted zinc finger unique to the DME/ROS1 family (see Fig. 1, a). Unlike in all other HhH superfamily DNA glycosylases, the catalytic domain in DME/ROS1 proteins is disrupted by a long non-conserved insert (Ponferrada-Marín et al., 2011). Based on the literature data on the AtROS1 protein, we previously cloned the NtROS1 cDNA fragment encoding amino acid residues 754–1796 (Gruber et al., 2018). This region contains all elements necessary for the catalytic activity in AtROS1 (Hong et al., 2014).

Since the structures of the DME/ROS1 family proteins are currently unknown, for a more detailed understanding of the organization of the NtROS1 catalytic fragment, we carried out homology modeling based on the AtDME and AtROS1 models from the AlphaFold template collection (Jumper et al., 2021). The two resulting models were almost identical except for the structure of the long non-homologous regions. The disrupted HhH domain folded into the α-helical structure characteristic of DNA glycosylases of this superfamily, in which a DNA binding groove with the catalytic residues Lys1341 and Asp1359 is evident (see Fig. 1, b). In addition, several more peripheral α-helices buttress the HhH domain and the FeS cluster and are obviously important for maintaining their structure. The FeS cluster, zinc finger, and RRM motif form separate structural elements that leave free access to the DNA binding groove (see Fig. 1, b). All disordered regions of the structure are also located on the side of the protein globule opposite to the DNA binding groove.

**Fig. 2.**
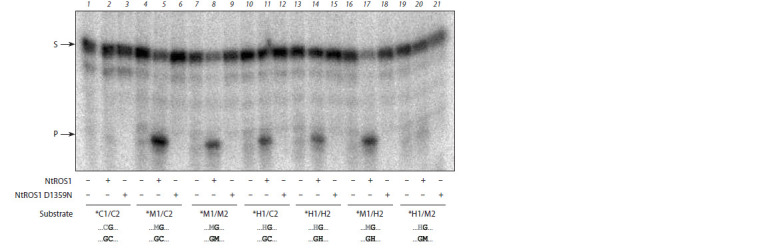
Cleavage of substrates (50 nM) with wild-type NtROS1 and NtROS1 D1359N (100 nM) for 60 min. The methylation context of the CpG site in each substrate is indicated below the gel image. Lanes 1, 4, 7, 10, 13, 16, and 19, substrates without the enzyme; lanes 2, 5, 8, 11, 14, 17, and 20, wild-type NtROS1; lanes 3, 6, 9, 12, 15, 18, and 21, NtROS1 D1359N. Arrows mark the substrate (S) and the cleavage product (P). In the schematic representations of substrates below the gel, the excised base is shown in gray.

To analyze the catalytic activity and substrate specificity of NtROS1, we performed a cleavage reaction of double-stranded oligonucleotides containing a CpG dinucleotide in which the cytosine was unmethylated, methylated, or hydroxymethylated in one or both chains (Fig. 2). The strand to be cleaved was 32P-labeled at the 5′-end. NtROS1 showed virtually no activity on the substrate containing an unmethylated CpG site, which is consistent with the literature data claiming that the C5 position of the cytosine must carry a substitution to be cleaved by AtROS1 (Morales-Ruiz et al., 2006). Activity towards mC and hmC was observed; however, its level differed markedly for both types of substrates. Comparing the efficiency of cleavage of M1/C2 and M1/M2 substrates (see Fig. 2, lanes 5 and 8) with H1/C2 and H1/H2 substrates (lanes 11 and 14), one can see that the enzyme prefers to excise mC over hmC both from CpG sites modified at only one strand and from fully modified sites. Small differences in the cleavage of M1/C2, M1/M2 and M1/H2 substrates (lanes 5, 8, and 17) indicate that modifications in the complementary strand have little effect on the removal of mC, and hmC in the complementary strand might even increase the cleavage. The enzyme showed no activity against DNA substrates containing uracil or 8-oxoguanine. The NtROS1 D1359N mutant, as expected, exhibited no glycosylase activity against any substrate with various combinations of mC and hmC, which confirms the importance of the Asp residue in the ROS1 catalytic center for the removal of modified bases from DNA.

**Fig. 3.**
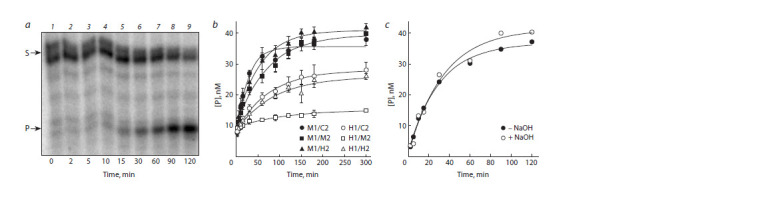
Time course of the hydrolysis of various substrates by NtROS1 a, Representative gel showing the accumulation of the reaction products after cleavage of the M1/M2 substrate for 0–120 min; b, cleavage of the M1/C2, M1/M2, M1/H2, H1/C2, H1/M2 and H1/H2 substrates (mean ± S.D. for 3–4 experiments are shown); c, cleavage of the M1/M2 substrate without and with NaOH treatment for the complete elimination of AP sites.

AtROS1 was reported to be an enzyme with a very low turnover number (Ponferrada-Marín et al., 2009), so it was not feasible to use steady-state kinetics to characterize the activity of NtROS1. To determine the apparent reaction rate constant, we used the conditions close to the single-turnover kinetic regime ([E]0 > [S]0). Under these conditions, all DNA substrate rapidly binds to the enzyme, and the reaction rate is limited not by substrate binding or product release, but by the chemical step of the pseudo-first order reaction, the hydrolysis of the N-glycosidic bond of the modified nucleotide (Porello et al., 1998). Thus, following the accumulation of the product over time (Fig. 3, a), one can estimate the reaction rate constant. The time courses for all substrates are shown in Fig. 3, b, and the rate constants calculated from these data are summarized below:

Substrate k·104, s−1 *M1/C2 6.5 ± 1.1 *M1/M2 2.4 ± 0.5 *M1/H2 3.0 ± 0.7 *H1/C2 1.9 ± 0.3 *H1/M2 1.6 ± 0.6 *H1/H2 2.0 ± 0.3 *C1/C2 Not cleaved * 32P-labeled strand.

In all cases, hmC was a worse substrate for NtROS1 compared to mC. Based on these results, we conclude that NtROS1 cleaves hemimethylated CpG sites most efficiently, while hmC in the context of HG/GM, on the contrary, is the worst substrate for this enzyme. The kinetic constants are consistent with the qualitative data on the relative cleavage efficiency for different substrates (see Fig. 2).

Many slow-turnover DNA glycosylases have lower AP lyase activity in comparison with their DNA glycosylase activity. In this case, after the removal of the modified base, a part of the reaction product exists as an AP site for a long time, and the true amount of the product can be revealed only upon treatment with alkali or nucleophilic amines (Porello et al., 1998). However, in the case of NtROS1, additional treatment with NaOH did not lead to a noticeable increase in the accumulation of the reaction product (see Fig. 3, c). Apparently, the rate of the reaction catalyzed by NtROS1 is limited by the hydrolysis of the N-glycosidic bond, which was also suggested for the enzyme from A. thaliana (Hong et al., 2014).

**Fig. 4.**
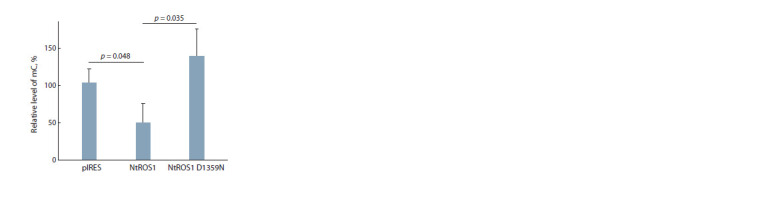
Relative amounts of mC in the DNA of HEK293 Phoenix cells transfected with the pIRES-eGFP-puro plasmid carrying no insert or carrying an insert encoding the NtROS1 catalytic fragment or its inactive variant NtROS1 D1359N. Mean ± S.D. of 3 experiments are shown.

To assess the ability of the NtROS1 protein to act as a demethylase when expressed in mammalian cells, we constructed plasmids based on the pIRES-eGFP-puro vector encoding wild-type NtROS1 and its catalytically inactive mutant NtROS1 D1359N. Using anti-mC antibodies, we have estimated the level of this epigenetic base in HEK293 cells after transfection with these plasmids. When cells were observed post-transfection, the proliferation of cells with the pIRES-eGFP-puro-NtROS1 plasmid was reduced by about 30 % compared to the control cells transfected with the plasmid with no insert and the cells transfected with the plasmid encoding the catalytic mutant. The fraction of live cells was the same in all cases, which indicates that the cell cycle may be slower in the presence of active NtROS1 due to the need to repair a large number of breaks introduced into DNA at mC residues. An analysis of the mC level revealed a ~2-fold decrease relative to control samples when wild-type NtROS1 was expressed and the absence of statistically significant changes with the expression of NtROS1 D1359N (Fig. 4). In general, it can be considered that the transient expression of the catalytic domain of 5-methylcytosine–DNA glycosylase NtROS1 in human cells indeed leads to the global erasure of mC epigenetic marks. The amount of hmC in cells could not be measured using anti-hmC antibodies, probably because this modified base is about an order of magnitude less abundant than mC (Yu et al., 2012; Zahid et al., 2016).

Thus, it can be concluded that NtROS1 is a DNA glycosylase specific for 5-methylcytosine and, to a lesser extent, for 5-hydroxymethylcytosine. Its biological functions, as in the case of AtROS1, most likely consist in the regulation of methylation status and gene expression during embryonic development (Yamamuro et al., 2014) or in the response to infections and abiotic stress (Gong et al., 2002; Le et al., 2014), and the regulation of silencing of transgenes and transposable elements (Gong et al., 2002; Kapoor et al., 2005; Zhu et al., 2007). The mechanism of RNA-dependent addressing of DME/ROS1 family proteins to specific demethylation sites, which has not yet been elucidated, is of great interest. The RRM motif in these polypeptides is homologous to the motifs responsible for nonspecific interactions with RNA in many other proteins (Cléry et al., 2008) and, by its position in the structure (see Fig. 1, b), could bind small RNAs complementary to the DNA stretch 5′ of the targeted mC. Zinc fingers of the CXXC type are used by many mC-recognizing proteins; however, they predominantly bind unmethylated DNA and are presumably required for accurate positioning of the enzyme in the presence of several methylation sites located at a short distance (Iyer et al., 2011).

The prospects for using NtROS1 and other proteins of the DME/ROS1 family as tools for genetic technologies largely depend on the possibility of reducing the size of the catalytic fragment. A deletion of the long insert between the two parts of the HhH domain in AtROS1 fully preserves its activity, but a deletion of the C-terminal tail after the FeS cluster results in an inactive enzyme (Hong et al., 2014). Judging from the structural models of AtROS1 and NtROS1, the HhH and RRM domains interact with each other, and shortening the protein here may only be done by trimming the insert between them. In any case, DME/ROS1 proteins, including NtROS1, represent a promising scaffold for enzyme engineering to analyze epigenetic methylation status and control gene activity.

## Conclusion

The study of the demethylating DNA glycosylase ROS1 from Nicotiana tabacum reported in this work presents the only biochemical investigation of ROS1 beyond its homologue from Arabidopsis thaliana. In addition to 5-methylcytosine, NtROS1 showed the ability to remove 5-hydroxymethylcytosine from DNA, but the efficiency of this reaction was lower than for 5-methylcytosine, apparently because plants rarely if at all use 5-hydroxymethylcytosine as an epigenetic marker. The observed decrease in global methylation upon expression of NtROS1 in human cells suggests that this protein or its optimized variants can be used as a tool for epigenetic regulation, either on its own or as an active module in constructs targeted to certain genome regions.

## Conflict of interest

The authors declare no conflict of interest.
